# Comparison of flexible ureteroscopy and mini-percutaneous nephrolithotomy in the treatment for multiple nephrolithiasis

**DOI:** 10.3389/fsurg.2022.1004432

**Published:** 2022-09-09

**Authors:** Guangda Lv, Zhiwei Zhang, Fei Du, Wenqiang Qi, Minglei Zhong, Yongheng Zhou, Changkuo Zhou, Yan Li, Dongqing Zhang

**Affiliations:** ^1^ Department of Urology, Qilu Hospital of Shandong University, Jinan, China; ^2^ Department of Urology, Yanggu People’s Hospital, Yanggu, China

**Keywords:** flexible ureteroscopy, mini-percutaneous nephrolithotomy, upper urinary tract calculi, propensity score matching, lithotripsy

## Abstract

**Objective:**

To compare the outcomes of flexible ureteroscopy and mini-percutaneous nephrolithotomy in the treatment for multiple nephrolithiasis in 1–2 cm size.

**Methods:**

The clinical data of patients with multiple renal calculi in the range of 1–2 CM who underwent flexible ureteroscopy lithotripsy and percutaneous nephrolithotomy in Qilu Hospital of Shandong University from January 2016 to March 2021 were retrospectively collected and matched using propensity score matching. Then a subgrouping of the number of stones was performed. Patients were divided into Group A and Group B according to their stone numbers. Patients with no statistically significant differences in baseline data were matched to compare the safety and efficacy of the two procedures.

**Results:**

A total of 210 patients with clinical data were collected, and the patients’ baseline data were not comparable, and 142 patients were finally included in the study after propensity score matching. There was no statistical difference in baseline data between the two groups of patients. The postoperative hospital days (3.00, 2.00 vs. 7.00, 3.00, *P* < 0.001), operation time (90.00, 50.00 vs. 110.00, 53.00, *P* = 0.018), complications (6, 6.8% vs. 14, 25.9%, *P* = 0.001) of patients in flexible ureteroscopy group %, *P* = 0.001) was significantly lower than that in the percutaneous nephrolithotomy group. There was no significant difference in stone clearance rate between the two groups (76, 86.4% vs. 42, 77.8%, *P* = 0.185). When the number of stones was no more than 3, the operation time (85.00, 49.00 vs. 110.00, 53.00, *P* = 0.005) and complications (2, 4.2% vs. 11, 29.7%, *P* = 0.001) of *f*-URS were significantly less than those of mPCNL, but when the number of stones was more than 3, there was no significant difference between the two operations.

**Conclusion:**

For multiple nephrolithiasis within 1–2 CM, when the number of stones does not exceed 3, flexible ureteroscopy can achieve the same stone clearance rate as percutaneous nephrolithotomy, while having shorter post-operation days, operative time and fewer complications. When the number of stones is more than 3, there are no significant difference between two operations.

## Introduction

In recent decades, the incidence of urolithiasis has been increasing worldwide, which has reached the highest incidence of 19% (1). The postoperative recurrence rate is high, which brings a heavy burden to patients and society. With the development of intracavitary lithotripsy technology, extracorporeal shock wave lithotripsy (SWL), retrograde intrarenal surgery (RIRS), and percutaneous nephrolithotomy (PCNL) have replaced traditional open lithotripsy and become the main treatment for urolithiasis (2). The European Association of Urology (EAU) guidelines pointed out that PCNL is the preferred surgical method for nephrolithiasis >2 CM, because it has the advantage that the surgical effect will not be affected by the length of the stone (3); for 1–2 CM nephrolithiasis, RIRS can be the first choice as it could achieving a stone-free rate (SFR) similar to PCNL with fewer complications (4). However, the evidence on the optimal treatment of multiple ipsilateral calculi is limited, the current guidelines do not provide clear recommendations, and many studies have investigated the effects of RIRS and PCNL on single calculi, with few studies on multiple calculi (5).

Arif et al. use stone surface area, which is more complicate and more difficult to access, instead of stone length, which is considered to be a golden standard variability to determine stone burden by many Guidelines (2), as a preoperative variable to measure stone burden (6). Ozer et al. included huge stones that exceed 3 CM, which were suitable for PCNL by guidelines (7). We use stone length to measure stones to comply with the guidelines. This study compared the efficacy and safety of flexible ureteroscopy (*f*-URS) and mini percutaneous nephrolithotomy (mPCNL) in the treatment of multiple kidney stones with a length of 1–2 CM, To better define the effect of stone number on the outcome of the two procedures, we performed a subgroup analysis based on stone number, which to our knowledge is the first time in studies of multiple stones.

## Materials and methods

### Study population and design

We retrospectively counted the clinical data of patients with multiple nephrolithiasis who received *f*-URS and mPCNL in Qilu Hospital of Shandong University from 2016.01 to 2021.03. Inclusion criteria were: (1) Multiple kidney stones with or without ureteral stones; (2) Stone length between 1–2 CM. The exclusion criteria were: (1) Patients with incomplete perioperative clinical data; (2) Patients who underwent bilateral lithotripsy during surgery; (3) Patients who underwent surgery for other diseases during hospitalization; (4) Patients who underwent other types of surgery during surgery; (5) Minor patients who were younger than 18 years old; (6) Patients with the presence of congenital anomalies of the upper urinary tract.

All patients were diagnosed with multiple kidney stones by Computed Tomography (CT), Kidneys, Ureter, and Bladder x-ray (KUB), Intravenous urography (IVU), ultrasound or other imaging data before operation. Patients who did not undergo CT examination were routinely performed CT examination before operation to identify stone characteristics. Routine examinations of blood, urine, liver and kidney function, blood biochemistry, and urine culture were performed in all patients before operation. Patients with positive urine culture should be treated with corresponding sensitive antibiotics for 5–7 days before surgery according to the culture results. All patients were treated with prophylactic antibiotics during the perioperative period.

We collected the patients' gender, age, American anesthesiologist classification (ASA), body mass index (BMI), stone burden, stone length, lower pole stones, Seoul national university renal stone complexity scoring system (S-SeRc), preoperative urine culture, postoperative hospital days, operation time, complications, SFR. We used ASA to assess the patient's preoperative physical status. Stone length is defined as the longest diameter of the largest of the stones, not the sum of the lengths of all stones taken by other studies using multiple stones. Because the purpose of our work was to investigate the effect of *f*-URS vs. mPCNL treating multiple renal stones with similar maximum stone lengths. Stone burden was calculated using the following formula to calculate the maximum cross-section of the stone: stone length * stone width * 3.14 * 0.25 (8). Therefore, the stone burden is also the largest cross-sectional area of a stone. We used the S-ReSc score to assess the number of pelvises and calyces occupied by stones (9). The operation time was defined as the time from the insertion of the rigid ureteroscope to the end of the operation. Complications were graded using the Clavien-Dindo grading system (10), and if two grades of complications were combined at the same time, it was defined as the highest grade. Immediate SFR was assessed 2 or 3 days after surgery using KUB. SFR after 3 month of the operation was also evaluated by KUB in outpatient clinic. Completely clear stones or residual stones ≤4 mm were defined as achieving the SFR criteria.

### Surgical procedure

#### Flexible ureteroscope lithotripsy

After the patient was under general anesthesia, take the lithotomy position, enter the rigid ureteroscope, find the ureteral orifice on the side with the stone in the bladder, and place the safety guide wire into the ureteral orifice until the renal pelvis under the watch of the rigid endoscope. If the patient's ureter is too thin to insert a flexible ureteroscope, a ureteral stent is placed to dilate the ureter, and lithotripsy is performed after 2 weeks. After that, the rigid ureteroscope was withdrawn, the 14/16 Fr UAS was placed along the safety guide wire, and then the safety guide wire was pulled out, and the flexible ureteroscope was inserted to reach the place with stones. After the stones were found by exploration, a 200 um holmium laser fiber was injected, the power was adjusted to 0.5–0.8 J, and the frequency was 10–20 Hz. After the lithotripsy is complete, the residual stone is removed from the body using a set of stone baskets. After the stones were cleared, a safety guide wire was inserted, and all patients were placed in a 6 Fr ureteral stent, which will be pulled out after 1 month if no ureteral injuries occurred in procedures.

#### Mini percutaneous nephrolithotomy

After successful anesthesia, the patient first takes the lithotomy position, routinely disinfects the perineum, and lays sterile surgical drapes into the ureteroscope into the bladder, find the ureteral orifice, put a 5F stent tube through the ureteral orifice, external 0.9% Sodium Chloride Solution irrigation, indwelling catheter, and fix the stent on the catheter. The patient was changed to the prone position, the lower back was routinely sterilized, and a sterile surgical drape was laid. The middle and posterior renal calyces of the kidney were punctured by ultrasound positioning, the urine was seen flowing out of the posterior guide wire, and the fascia dilator was gradually expanded to F16 along the guide wire, and the ureteroscope was inserted. If there are multiple stones in the cup, the ureteroscope is removed, the rigid nephoscope is replaced, and the stones are gradually crushed and flushed out by the holmium laser. After checking no obvious stones and bleeding, a 6F double “J” tube was indwelled by an ultra-smooth guide wire. The nephoscope was withdrawn, and a 14F nephrostomy tube was indwelled and fixed with silk thread.

### Statistical analysis

Propensity score matching (PSM) was performed using RStudio (Version 1.4.1106), indicators included gender, age, stone burden, stone length, ASA, preoperative urine culture, lower pole stones, caliper value = 0.05, radio = 2. Data were statistically analyzed using IBM SPSS Statistics Version 25 (International Business Machines Corporation, Armonk, New York, NY, USA). Shapiro-Wilk-Test was used to identify whether continuous variables conform to a normal distribution or not. If continuous variables do not conform to a normal distribution, use the median and interquartile range to express, and if they conform to a normal distribution, use the mean and standard deviation to express, and the Wilcoxon–Mann–Whitney-Test was used to analyze the differences of binary variables. Dichotomous variables were expressed as percentages, and differences were analyzed using the chi-square test. *P* < 0.05 means there is a statistical difference.

## Results

A total of 210 patients were counted and divided into *f*-URS group and mPCNL group according to the different operations they received, 140 were in the *f*-URS group and 70 in the mPCNL group. 1 patient had recurrence of stones after a lapse of 1 year, and this patient was treated with *f*-URS in both operations. There was a statistically significant difference in stone length between the two groups, so PSM was used to identify patients with no statistical difference in preoperative clinical data, and 142 patients were finally included in the study. There were 54 patients in the mPCNL group and 88 patients in the *f*-URS group. There was no statistical difference in preoperative clinical data between the two groups of patients.

All patients underwent surgery successfully, and a total of 6 underwent secondary surgery. Among them, there were 2 patients in the mPCNL group, one underwent mPCNL again, the other underwent dual-scope combined lithotripsy. Four patients in the *F*-URS group underwent secondary operations, and the secondary operations were all *f*-URS. 5 patients developed postoperative fever, including 4 patients in mPCNL group and 1 patient in *f*-URS group; 8 patients developed systemic inflammatory response syndrome (SIRS) after surgery, including 6 patients in mPCNL group and 2 patients in *f*-URS group; The above complications were grade1. There were 6 patients with postoperative sepsis, including 4 patients in mPCNL group and 2 patients in *f*-URS group; the above complications were grade 2. One patient in the mPCNL group underwent laparoscopic nephrectomy due to postoperative renal failure, bleeding and other complications, and the complication was grade 3b. One patient in the *F*-URS group developed postoperative pulmonary embolism and was transferred to ICU for monitoring and treatment. mPCNL had greater postoperative hospitalization days (7.00, 3.00 vs. 3.00, 2.00, *P* < 0.001), operation time (110.00, 53.00 vs. 90.00, 50.00, *P* = 0.018) and complications (14, 25.9% vs. 6, 6.8%, *P* = 0.001) compared with *f*-URS group. However, the SFR between the two groups was not significantly different (42, 77.8% vs. 76, 86.4%, *P* = 0.185) (Table 1).

**Table 1:** Comparison of the clinical characteristics of included patients.

	Before PSM (*n* = 210)			After PSM (*n* = 142)		
	mPCNL (*n* = 70)	f-URS (*n* = 140)	*P*	mPCNL (*n* = 54)	f-URS (*n* = 88)	*P*
Sex (*n*, %)			0.175			0.275
Male	43 (61.4%)	99 (70.7%)		38 (70.4%)	54 (61.4%)	
Female	27 (38.6%)	41 (29.3%)		16 (29.6%)	34 (38.6%)	
Age (M, Q)	52.00, 21.00	52.00, 15.00	0.420	51.00, 24.00	53.00, 15.00	0.916
BMI (kg/m^2^, X ± S)	26.25 ± 3.75	25.97 ± 3.45	0.595	26.69 ± 3.91	26.18 ± 3.53	0.422
ASA			0.392			0.795
I	6	17		6	11	
II	64	123		48	77	
Stone burden (mm^2^,M,Q)	1.18, 0.98	1.18, 1.08	0.782	1.20, 0.94	1.18, 1.29	0.961
Stone length (cm, M, Q)	1.50, 0.60	1.50, 0.80	0.038*	1.50, 0.63	1.50, 1.00	0.860
Lower pole stone	42 (60.0%)	94 (67.1%)	0.307	34 (63.0%)	58 (65.9%)	0.721
S-ReSc			0.553			0.450
Low	24	37		19	26	
Middle	33	81		28	48	
Heavy	13	22		7	14	
Urine culture (*n*, %)	12 (17.1%)	15 (10.7%)	0.190	6 (11.1%)	12 (13.6%)	0.661
Post-operation days (day, M, Q)	7.00, 2.00	3.00, 2.00	*P* < 0.001	7.00, 3.00	3.00, 2.00	*P* < 0.001*
Operation time (min, M, Q)	107.50, 51.00	90.00, 55.00	0.011	110.00, 53.00	90.00, 50.00	0.018*
Complication (*n*, %)	21 (30.0%)	11 (7.9%)	*P* < 0.001	14 (25.9%)	6 (6.8%)	0.001*
I	16	7		10	3	
II	5	3		4	2	
IIIb	1	0		1	0	
IV	0	1		0	1	
Immediate SFR (*n*, %)	55 (78.6%)	122 (87.1%)	0.108	42 (77.8%)	76 (86.4%)	0.185
SFR after 3 month (*n*, %)	67 (95.7%)	135 (96.4%)	1.000	52 (96.3%)	84 (95.5%)	1.000

* *P* < 0.05, which means statistically significant.

When the number of stones was no more than 3, the operation time (85.00, 49.00 vs. 110.00, 53.00, *P* = 0.005) and complications (2, 4.2% vs. 11, 29.7%, *P* = 0.001) of *f*-URS were significantly less than those of mPCNL, but when the number of stones was more than 3, there was no significant difference between the two operations (Table 2).

**Table 2:** Subgroup of the number of stones.

	Group A			Group B		
	mPCNL (*n* = 37)	f-URS (*n* = 48)	*P*	mPCNL (*n* = 17)	f-URS (*n* = 40)	*P*
Post-operation days (day, M, Q)	7.00, 3.00	3.00, 2.00	*P* < 0.001*	7.00, 3.00	3.00, 1.00	*P* < 0.001*
Operation time (min, M, Q)	110.00, 53.00	85.00, 49.00	0.005*	105.00, 43.00	97.50, 65.00	0.787
Complication (*n*, %)	11 (29.7%)	2 (4.2%)	0.001*	3 (17.6%)	4 (10.0%)	0.716
Immediate SFR (*n*, %)	32 (86.5%)	43 (89.6%)	0.920	10 (58.8%)	33 (82.5%)	0.118
SFR after 3 months (*n*, %)	36 (97.3%)	46 (95.8%)	1.000	16 (94.1%)	38 (95.0%)	1.000

* *P* < 0.05, which means statistically significant.

## Discussion

Since its introduction in the 1970s, percutaneous nephrolithotomy has gradually replaced the traditional open lithotomy as the main treatment for urolithiasis due to its advantages of high SFR and less trauma. Its advantage is that the SFR is not limited by the length of the stone, which makes it competent for almost all types of complex kidney stones, including staghorn stones (11). With the development of intracavity lithotripsy equipment, PCNL has also developed new forms. In order to reduce complications such as bleeding and kidney injury caused by puncture, PCNL has developed mini-PCNL with nephroscope between 14 and 18 Fr and ultra-micro channel PCNL and micro-PCNL with nephroscope less than 14 Fr from the standard PCNL which has traditional nephroscope of 22–26 Fr. (12–14). Ureteroscopy lithotripsy technology has also developed rapidly with the development of equipment. Semi-rigid ureteroscope and soft ureteroscope have been developed from the initial rigid ureteroscope. Flexible ureteroscope can reach any renal pelvis and calyces by virtue of its multi-bending feature. With the development of laser generators, *f*-URS can currently treat kidney stones in any location (15). *f*-URS reaches the surgical site through the body's natural channels, and has good protection for blood vessels and kidneys (5), and is favored by more and more doctors and patients. However, the current surgical effect of *f*-URS is still affected by the length of the stone. For kidney stones >2 cm, the EAU guidelines still recommend PCNL as the first choice. For 1–2 cm kidney stones, the guidelines believe that *f*-URS can achieve a similar SFR as PCNL with lower complications (16–18). However, there is still a lack of evidence on the optimal management of ipsilateral multiple stones within 1–2 cm. This study retrospectively compared the outcomes of *f*-URS and mPCNL in the treatment of multiple nephrolithiasis within 1–2 cm, and performed a subgroup analysis based on stone number.

Few studies have reported the surgical effect of *f*-URS and mPCNL in the treatment of multiple nephrolithiasis within 1–2 cm. However, there are many studies on the prognostic factors of stone removal surgery. Kozyrakis D et al. found that neither the number nor location of stones had a significant effect on the outcome of lithotripsy (19), so physicians should not discourage the use of *f*-URS in patients with multiple kidney stones. Studies showing that the number of stones affects the outcome of surgery are mostly focusing on SWL (20). Our results show that the effect of *f*-URS and mPCNL in the treatment of multiple nephrolithiasis is similar to those of single nephrolithiasis. *f*-URS has a similar SFR as mPCNL, while having lower complications, operative time and postoperative, postoperative hospital days.

Previous studies have shown that the incidence of SIRS after PCNL ranges from 16.7% to 23.4% (21, 22), while the incidence of SIRS after surgery in this study, including those who developed sepsis, was 18.5%. In contrast, the incidence of postoperative infection after RIRS ranged from 1.7% to 18.8% (23), and the incidence in this study was 2.2%. As an invasive operation, PCNL inevitably requires a puncture operation. Although the 16/18Fr microchannel has significantly reduced the probability of bleeding compared with the 26 Fr standard channel, it still cannot avoid damage to the renal parenchyma or blood vessels, which may be the reason why the complications of mPCNL are generally higher than those of *f*-URS. Age, stone length, small diameter UAS, and excessive perfusion pressure are considered to be independent risk factors for complications after *f*-URS (23). In this study, 14/16 Fr UAS was used, which was a larger diameter that made the perfusion fluid flow out easily to reduce the pressure in the renal pelvis, and we paid close attention to the perfusion pressure in the renal pelvis during the perfusion process. Those may be the reasons for the lower complication rate after *f*-URS in our study.

The SFR of mPCNL in this study was 77.8%, which was lower than that of other studies, which may be related to the earlier time of our SFR assessment. But the SFR after 3 months of operation achieved to 97.3%. In most studies, SFR was assessed at 1 month or 3 months after surgery. However, different activity status of patients after surgery may have biased the results. Lee S H et al. found independent risk factors for immediate postoperative SFR, but no independent risk factors for SFR after one month of the surgery was found (24), indicating that unknown activity status after surgery will affect SFR, so it cannot be accurately assessed effect of surgery. Therefore, we used the immediate postoperative SFR as the outcome to evaluate the surgical effect.

This study was a retrospective study, and the sample size was further lost after the use of PSM to a small sample size, which did not include staghorn stones after matching. Although most variables were not statistically different, the most important variable, stone length, had a large different distribution between two groups, so we had to use PSM to obtain comparable patients. In this study, ASA was used to describe the preoperative status of patients, the results may be biased.

## Conclusion

We compared the efficacy of PCNL vs. *f*-URS in the treatment of multiple kidney stones within 1–2 cm. And subgroup analysis was performed according to the number of stones. When the number of stones did not exceed 3, the operation time of *f*-URS was significantly shorter than that of the mPCNL, and the complication was also lower than that of the mPCNL. When the number of stones was more than 3, there is no significant difference in the operation time or complications between the two operations. Regardless of the number of stones, there was no difference in stone-free rate between the two surgical modalities, and *f*-URS was associated with less post-operation days than PCNL. Above all, *f*- URS shows better efficacy when treating multiple nephrolithiasis within 1–2 cm, especially for the number of stones no more than 3. *F*-URS could achieve an equal performance to mPCNL even if the number of stones exceeds 3.

## Data Availability

The raw data supporting the conclusions of this article will be made available by the authors, without undue reservation.
